# 2019 Novel Coronavirus Disease (COVID-19): Paving the Road for Rapid Detection and Point-of-Care Diagnostics

**DOI:** 10.3390/mi11030306

**Published:** 2020-03-14

**Authors:** Trieu Nguyen, Dang Duong Bang, Anders Wolff

**Affiliations:** 1 Department of Biotechnology and Biomedicine, Technical University of Denmark, 2800 Kongens Lyngby, Denmark; tring@dtu.dk; 2 Laboratory of Applied Micro and Nanotechnology (LAMINATE), Division of Microbiology and Production, National Food Institute, Technical University of Denmark. Kemitorvet, Building 204, 2800 Lyngby Denmark; ddba@food.dtu.dk

**Keywords:** COVID-19, Wuhan, 2019 novel Coronavirus, Point-of-care detection, SARS-CoV-2, (Loop-mediated isothermal amplification) LAMP assay, (polymerase chain reaction) PCR

## Abstract

We believe a point-of-care (PoC) device for the rapid detection of the 2019 novel Coronavirus (SARS-CoV-2) is crucial and urgently needed. With this perspective, we give suggestions regarding a potential candidate for the rapid detection of the coronavirus disease 2019 (COVID-19), as well as factors for the preparedness and response to the outbreak of the COVID-19.

## 1. Introduction

**Figure 1:**
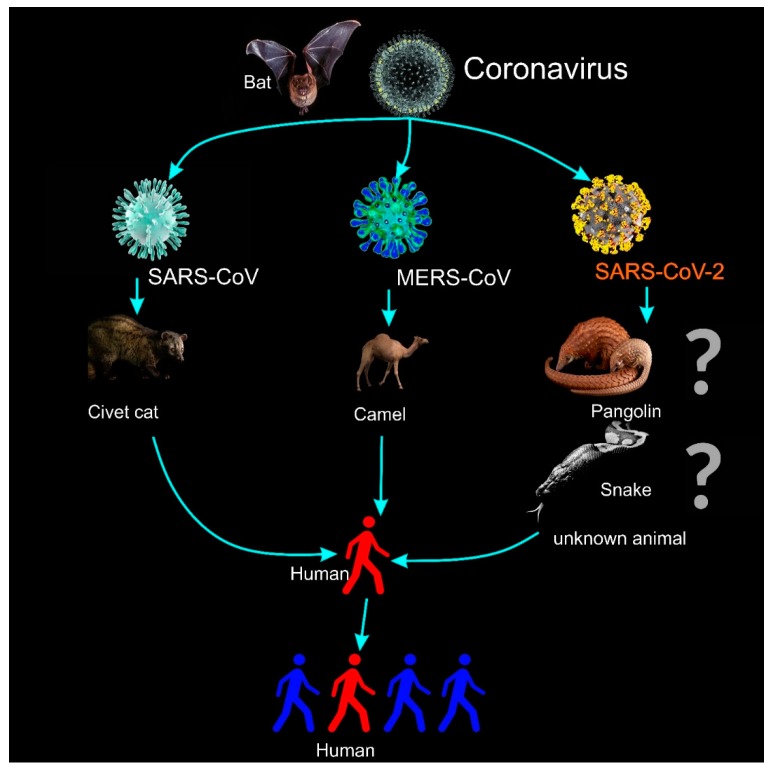
The illustration for the transmission of coronaviruses and the 2019 novel Coronavirus (2019-nCoV or SARS-CoV-2 [11]). Current studies have suggested that the intermediate carriers may be snakes [12] or pangolins [13], but according to WHO the real source is still unknown [14,15].

On 30 January 2020, the World Health Organization (WHO) declared a global public health emergency [1] over the outbreak of the new coronavirus, called the 2019 novel Coronavirus (2019-nCoV), which originated in Wuhan City, in the Hubei Province of China. On 11 February, WHO officially named the disease as the coronavirus disease 2019 (COVID-19) [2]. Human-to-human transmission (Figure 1) has been confirmed by WHO and by The Centers for Disease Control and Prevention (CDC) of the United States [3], with evidence of person-to-person transmission from three different cases outside China, namely in the US [4], Germany [5], and Vietnam [6]. COVID-19 has continuously spread to 104 countries; the number of confirmed infections reached 109,343 on 9 March 2020 [7], and the death toll in China has overtaken the SARS epidemic of 2002–2003 and has risen to 3,100 [2]. To slow down the spread of COVID-19, at least 50 million people in China have been placed under lockdown [8]. On 8 March 2020, Italy also undertook the same measures, with the northern part of the country placed under lockdown, affecting 16 million people [9]. The definition of coronaviruses is listed in Table 1. The reproduction number R_0_ (i.e., the average number of secondary cases generated by a typical infectious individual) is estimated to be 2.68, and the doubling time is estimated to be 6.4 days [10].

## 2. The Need for a Rapid Detection Method and Portable Detection Devices

The manifestation of the COVID-19 infection is highly nonspecific, including respiratory symptoms, fever, cough, dyspnea, and viral pneumonia [20]. Thus, diagnostic tests specific to this infection are urgently required to confirm suspected cases, screen patients, and conduct virus surveillance.

**Figure 2:**
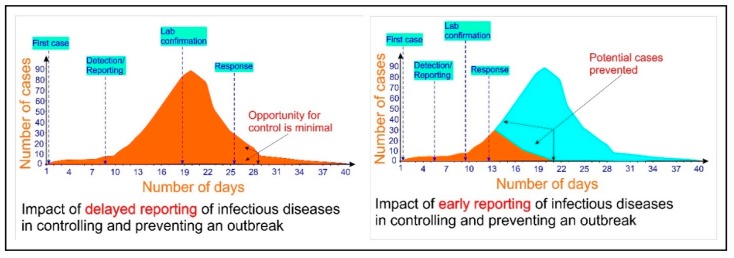
The dramatic impact of the rapid detection of infectious diseases in controlling and preventing an outbreak (adapted to WHO document [22] and References [23,24]).

In this scenario, a point-of-care (PoC) device (i.e., a rapid, robust, and cost-efficient device that can be used onsite and in the field, and which does not necessarily require a trained technician to operate [21]) is crucial and urgently needed for the detection of COVID-19. Figure 2 shows the dramatic impact of early detection of infectious diseases in controlling an outbreak [22,23,24]. 

Such a PoC device can be used in (but is not limited to) an emergency situation, such as the Diamond Princess cruise ship case. Recently, it was reported that the Diamond Princess cruise ship has been quarantined in Yokohama, Japan, due to a serious spreading of COVID-19 on this cruise, with at least 454 infected cases out of 3,700 passengers and crew (reported by WHO [7], 17 February 2020). The detection of COVID-19 may not have been prompt enough as they did not have enough test kits to diagnose all the passengers on the ship in order to timely respond to the rapid spreading of the disease [25].

The current standard molecular technique that is now being used to detect COVID-19 is the real-time reverse transcription-polymerase chain reaction (rRT-PCR). This protocol has been documented and available online on the WHO website since 17 January 2020 [26]. The testing procedure includes: (i) specimen collection; (ii) packing (storage) and shipment of the clinical specimens; (iii) (good) communication with the laboratory and providing needed information; (iv) laboratory testing; (v) reporting the results. This rRT-PCR technique requires sophisticated laboratory equipment that is often located at a central laboratory (biosafety level 2 or above) [4,26,27]. Sample transportation is inevitable. As a consequence, the time required to obtain the results can be up to 2 or 3 days. In the case of a public health emergency such as the COVID-19 outbreak, this time-consuming process of sample testing is not only extremely disadvantageous, but also dangerous since the virus needs to be contained. In addition, commercial PCR-based methods are expensive and depend upon technical expertise, and the presence of viral RNA or DNA does not always reflect acute disease [28,29,30]. Furthermore, using PCR, codetection with other respiratory viruses is frequently encountered in coronaviruses (CoVs), and the contribution of positive CoV PCR results to disease severity is not always explicitly exhibited [28,29,30]. Furthermore, as of 2 February 2020 in the United States, as mentioned in the Interim Guidelines for Collecting, Handling, and Testing Clinical Specimens from Persons Under Investigation (PUIs) for 2019 Novel Coronavirus, the diagnostic testing for COVID-19 can be conducted only at CDC. From 4 February 2020 onwards, COVID-19 tests can also be done at laboratories designated by CDC. Likewise in China, where the outbreak is ongoing, samples had to be sent to Beijing for testing, as reported on 31 January 2020 [31]. On 4 February 2020, China’s own CDC deployed a mobile biosafety laboratory to Wuhan in the Hubei Province to assist with the response [32,33]. On 5 February 2020, an emergency test laboratory (biosafety level 2) run by BGI Genomics, Global Heartquare: Shenzhen, China was set up in Wuhan in the Hubei Province to assist the COVID-19 epidemic [34].

## 3. A Potential Candidate for Rapid Detection of SARS-CoV-2: Loop-Mediated Isothermal Amplification (LAMP) Assays in PoC Devices

In order to overcome the current time-consuming and laborious detection technique using RT-qPCR, an alternative molecular amplification technique should be deployed. Loop-mediated isothermal amplification (LAMP) reaction is a novel nucleic acid amplification technique that amplifies DNA with high specificity, efficiency, and rapidity under isothermal conditions. This method uses a set of four specially designed primers, and a DNA polymerase with strand displacement activity [35] to synthesize target DNA up to 10^9^ copies in less than an hour at a constant temperature of 65 °C. The final products are stem-loop DNAs with multiple inverted repeats of the target, bearing structures with a cauliflower-like appearance. LAMP has high specificity and sensitivity and is simple to perform; hence, soon after its initial development it became an enormously popular isothermal amplification method in molecular biology, with application in pathogen detection. LAMP uses strand-displacement polymerases instead of heat denaturation to generate a single-stranded template; hence, it has the advantage of running at a constant temperature, simultaneously reducing the cumbersomeness of a thermocycler as well as the energy required. LAMP technology is proven to be more stable [36] and more sensitive [37] in detection compared to PCR. Other advantages of LAMP compared to those of PCR are shown in Table 2. 

We believe that the LAMP assay could be a potential candidate for the point-of-care device application in the detection of COVID-19. An example of using LAMP in a point-of-care device for the detection of a zoonotic virus causing respiratory symptoms such as the Avian influenza virus (AIV) is the VIVALDI (Veterinary validation of point-of-care diagnostic instrument) project [38]. With PoC devices such as the VETPOD [38] (Veterinary, Portable, Onsite Detection), in the VIVALDI project, detection time can be less than 1 h. Besides PoC devices using disposable polymer chips and LAMP assays, as in the VETPOD of the VIVALDI project, a lateral flow strip (LFS) would also be a suitable candidate for the rapid and on-site detection of COVID-19. A device such as COVID-19 IgM/IgG Rapid Test of BioMedomics is a good example [39]. The sensitivity of the COVID-19 IgM/IgG Rapid Test is 88.66%, which is expected to be lower than the sensitivity of tests based on LAMP-reaction assays (>95%). Therefore, a combination of LFS and LAMP into one device could be an excellent candidate for PoC testing of COVID-19.

## 4. Other Important Factors in Fighting COVID-19

Furthermore, alongside detecting and containing the virus, for the sake of a public health response regarding the dynamics of the outbreak, the socio-economic impact of COVID-19 is equally in urgent need. WHO announced that to fight the further spread of COVID-19, the international community has launched a US$675 million preparedness and response plan from February through April 2020 [43]. In 2003, the SARS-CoV virus pulled the world’s output down by $50 billion. The early estimation for the cost to the global economy as a result of the outbreak of COVID-19 is about $360 billion [44]. This is because China’s GDP shares were approximately 17% globally as of 2019, which was about four times higher than in 2003, and the confirmed infected cases (at the time of doing the economic estimation, i.e., at the beginning of February 2020) are more than 2 times larger than the total of SARS. Given that the number of infected cases (109,343 confirmed cases) is currently approximately 14 times larger than SARS cases [45], and that the death toll due to COVID-19 has surpassed that of the SARS epidemic, the economic impact of COVID-19 might be much larger than $360 billion.

Furthermore, in order to win the battle against this outbreak, information on the epidemiological characteristics, such as the identification of the animal reservoirs [46] (Figure 1) and the risk factor of the disease, is also essential. The intermediate host carrying the disease is important to identify not only for the current epidemic, but also to eliminate a future outbreak. Together with the all aforementioned factors, the race for a vaccination against COVID-19 is equally essential. Although at this stage there is no registered treatment or vaccine for COVID-19, Zhang has recently mentioned some potential interventions [47], such as nutritional interventions (Vitamin A, B, C, D, E, and other trace minerals such as zinc and iron). Due to the high percentage of identicality in the sequence (up to 82% of the genome structure) between SARS-COV-2 and the SARS-CoV virus, immuno-enhancers and other specific treatment that have been applied for SARS could also be considered [47] to use for the treatment of SARS-CoV-2. 

## Figures and Tables

**Table 1:** Coronaviruses (CoVs) and 2019 novel coronavirus (SARS-CoV-2).

Virus	Description
Coronaviruses (CoVs):	A large and diverse family of enveloped, positive-stranded RNA viruses, with a ~26–32 kilobase genome [16]. The Coronaviridae cover a broad host range, infecting many mammalian and avian species, and induce upper respiratory, gastrointestinal, hepatic, and central nervous system diseases [17]. In the last few decades, coronaviruses have been shown to be capable of also infecting humans. The outbreak of severe acute respiratory syndrome (SARS) in 2003, and, more recently, Middle-East respiratory syndrome (MERS) have proved the lethality of CoVs when they cross the species barrier and infect humans [18].
2019 novel coronavirus (SARS-CoV-2 [11]):	A new zoonotic human coronavirus, which was reported and announced by the Chinese Center for Disease Control and Prevention (CCDC) on 9 January 2020 [19]. In spite of the fact that the initial infected cases have been associated with the Huanan South China Seafood Market, the source of COVID-19 is still unknown (Figure 1). On 30 January 2020, the WHO declared a global public health emergency regarding the outbreak of COVID-19. On the 11 March 2020, the WHO declared the outbreak of COVID-19 a pandemic.

**Table 2:** Comparison between PCR and loop-mediated isothermal amplification (LAMP) reactions [37,40,41,42].

PCR	LAMP
Thermal cycling (Multiple heating and cooling cycle; hence, bulky and cumbersome).	Isothermal and continuous amplification (Smaller, simpler, hence portable).
Always requires sample concentration and preparation (Time-consuming).	For virus detection, for example, influenza [40] or human norovirus, LAMP assay offers one-step detection [41]. Sample preparation steps are simplified.
Multiple protocols (Complicated and requires a skilled technician).	Single protocol (Faster).
Inhibitors hinder the reaction.	Tolerate inhibitors and more stable.
Diagnostic sensitivity (95%) is currently reported lower than LAMP [33,41,42].	Diagnostic sensitivity > 95%.
Established technique.	Applications using LAMP assays are being explored.
